# Modifying Effect of the Antibiotic Action, Membrane Permeability, and ADMET/Toxicity Profile of the Fixed Oil From the Pulp of *Mauritia Flexuosa* F.L. (Arecaceae)

**DOI:** 10.1002/cbdv.202503245

**Published:** 2026-04-15

**Authors:** Isaac Moura Araújo, Cleidiana Tomé de Oliveira, Luís Pereira‐de‐Morais, Andressa Silva Alencar, Cicera Datiane de Morais Oliveira‐Tintino, Maria Alícia Cavalcante Narciso, Anita Oliveira Brito Pereira Bezerra Martins, Sara Tavares de Sousa Machado, Sheila Alves Gonçalves, Daniel Sampaio Alves, Janaina Esmeraldo Rocha, Ana Carolina Ferreira Araújo, Priscilla Ramos Freitas, José Weverton Almeida‐Bezerra, José Thyálisson da Costa Silva, Henrique Douglas Melo Coutinho

**Affiliations:** ^1^ Department of Biological Chemistry Regional University of Cariri–URCA Crato Brazil; ^2^ Department of Biological Sciences Regional University of Cariri–URCA Crato Brazil

**Keywords:** antibacterial activity, buriti oil, *pseudomonas aeruginosa*, resistance, *staphylococcus aureus*

## Abstract

The increasing bacterial resistance to antibiotics has stimulated the search for natural products capable of modulating antimicrobial activity. In this study, the fixed oil from *Mauritia flexuosa* pulp was evaluated for its antibacterial activity, modulatory effect on antibiotics, membrane permeability, and ADMET/toxicity profile. The chemical composition of the oil was analyzed by GC–MS after transesterification, revealing fatty acids as the major constituents, particularly palmitic and elaidic acids. Antibacterial activity and modulation assays were performed using standard microdilution methods, while membrane permeability was assessed using SYTOX Green. The fixed oil showed no significant antibacterial activity under the tested conditions; however, it demonstrated a modulatory effect when combined with antibiotics, enhancing their activity. Membrane permeability assays indicated increased bacterial membrane disruption. In silico ADMET analysis suggested low systemic toxicity, with potential irritative effects. These findings indicate that *M. flexuosa* fixed oil may act as a modulator of antibiotic activity, possibly through membrane interaction mechanisms µg/mL.

## Graphical abstract

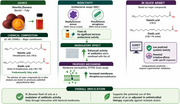

## 1 Introduction

Bacterial resistance is a process characterized by the loss of sensitivity of an antibiotic already used against a bacterium [1]. According to estimates by the World Health Organization (WHO), by 2050 the number of deaths due to infections caused by resistant microorganisms will be higher than the number of deaths caused by other diseases, due to the prescription and indiscriminate use of antibiotics. Among the main mechanisms of antibiotic resistance are changes in membrane permeability, changes in the site of action of the antibiotic, the production of enzymes that destroy antibiotics and the efflux mechanism [2].

*Staphylococcus aureus* is a bacterial strain that is currently widely studied, given its significant impact on public health, and is frequently found in microbial communities associated with a human or other animal host [3]. Although frequently found in the human microbiota, *S. aureus* can cause diseases ranging from simple skin infections to more serious diseases such as meningitis, endocarditis, toxic shock syndrome and septicemia [4, 5].

*Pseudomonas aeruginosa* is a Gram‐negative, nonlactose fermenting bacillus present in various environments, including soil and water. This species is characterized as an opportunistic microorganism and is considered one of the most important agents of hospital infections. The clinical importance of *P. aeruginosa* is related to the difficult eradication of the infection and continuous therapeutic failures, a consequence of the wide expression of virulence factors, as well as natural and acquired resistance to many antibiotics and disinfectants [6].

Natural products have been widely investigated as promising sources of bioactive compounds with antimicrobial properties. In particular, plant‐derived oils have attracted attention due to their complex chemical composition and potential to interact with biological membranes. Fixed oils, rich in fatty acids, have been associated with mechanisms such as membrane destabilization, increased permeability, and modulation of antibiotic activity [7]. These properties make them attractive candidates as adjuvants in antimicrobial therapy, especially against resistant strains.

*Mauritia flexuosa* L.F (buriti) is a palm tree of the Arecaceae family, native to the Amazon region, distributed throughout the states of Amazonas, Bahia, Ceará, Goiás, Maranhão, Minas Gerais, Pará, Piauí, Roraima, São Paulo, and Tocantins [8], Furthermore, it can also be found in other countries such as Bolivia, Guiana, Colombia, Venezuela, Trinidad, Ecuador, and Peru [9].

It is a plant whose fruit, stem, and leaves can be used for various purposes, from crafts to the pharmaceutical and food industries. The oil extracted from the pulp is used in cooking and folk medicine [10], Furthermore, it has aroused considerable interest due to the antimicrobial, anti‐inflammatory, healing, and antioxidant activities presented by its compounds [11].

Given this statement, the development of this study is justified, which is based on identifying the chemical composition and evaluating the antibacterial activity of *M. flexuosa* L.F against standard and multiresistant bacterial strains, such as the association of this compound with antibiotics. Therefore, the present study aimed to evaluate the antibacterial activity, modulatory effect on antibiotics, membrane permeability, and ADMET/toxicity profile of the fixed oil obtained from the pulp of *Mauritia flexuosa*. By integrating microbiological assays, membrane permeability analysis, and in silico predictions, this work seeks to provide new insights into the potential application of this natural product as an adjuvant in antimicrobial therapy.

## 2 Materials and Methods

### 2.1 Fixed Oil Extraction

The fresh fruits collected in Picos—PI were soaked in water to remove the scales. After that, the entire mesocarp (pulp) was removed and boiled in water at 60°C for approximately 20 min. After boiling, the oil was manually separated and extracted.

A voucher specimen was deposited in the “Herbário Caririense Dárdano de Andrade‐Lima—HCDAL” at the Regional University of Cariri—URCA under register number 12.620.

### 2.2 Microbiological Essays

#### 2.2.1 Minimum Inhibitory Concentration Test

The bacteria used in the study are part of the microorganism bank of the Regional University of Cariri—URCA, which were inoculated in BHI (Brain Heart Infusion Broth) and kept in a bacteriological incubator at 37°C/24 h. The microorganisms used were: *Staphylococcus aureus* 10 and *Pseudomonas aeruginosa* 24.

Microtubes were prepared, each containing 900 µL of 10% BHI and 100 µL of the bacterial suspension, previously diluted in saline solution until 0.5 of McFarland scale (1.5 x 10^8^ CFU/mL) was established. 100 µL of this solution added to each well (96‐well plate) and then serial microdilution was performed with a 100 µL solution of the OFFMF, varying in concentrations from 1024 to 8 µg/mL. The plates were placed in an incubator for 24 h at 35°C. The MIC was revealed using resazurin. The MIC is defined as the lowest concentration at which no growth was observed [12, 13]. All tests were performed in triplicate.

#### 2.2.2 Antibiotic Action‐Modifying Activity

The MIC of the antibiotics erythromycin, gentamicin, and norfloxacin was performed in the presence and absence of oil in sterile microdilution plates. OFFMF was tested at subinhibitory concentration (MIC/8). Then, 100 µL of the drugs erythromycin, gentamicin and norfloxacin at a concentration of 1024 µg/mL were mixed in the first well, proceeding to serial microdilution, in a ratio of 1:1 until the penultimate well. The concentrations of antimicrobials varied gradually from 1024 to 0.5 µg/mL [14, 15]. The microorganisms used were: multiresistant *Staphylococcus aureus* 10 and *Pseudomonas aeruginosa* 24. All tests were performed in triplicate (n = 3).

#### 2.2.3 Evaluation of Bacterial Membrane Permeability by the Fluorescence Method With SYTOX Green

In the permeability tests, *Staphylococcus aureus* and *Escherichia coli* were used as representatives of Gram‐positive and Gram‐negative bacteria, respectively. This choice allowed us to evaluate the effect of the oil on different cell wall types, including the lipopolysaccharide outer membranes typical of Gram‐negative bacilli such as *E. coli* and *Pseudomonas aeruginosa*.

For this test, the DNA‐intercalating dye SYTOX Green was used. A bacterial inoculum of *S. aureus* 10 and *E. coli* 06 strains was prepared. The inoculum was distributed in a 96‐well black plate. Fixed oil of *Mauritia flexuosa* (OFFMF) was added at final concentrations of 200 and 100 µg/mL. Polymyxin B was used as a positive control at a concentration of 200 µg/mL. Phosphate Buffer Saline (PBS) was added to the negative control group. The plates were incubated for 1 h. Then, 100 µL of SYTOX Green at a final concentration of 1 µM was added. The plates were incubated for 1 h, and fluorescence readings were performed using a Cytation 1 plate reader, BioTek (Winooski, VT, USA) and Gen5 3.11 Software. A 485 nm excitation and 528 nm emission filter were used [16]. The tests were performed in triplicate.

### 2.3 Phytochemical Characterization of the Fixed Oil

The identification of fatty acids present in the fixed oil of *Mauritia flexuosa* was performed through their respective methyl esters. The saponification of the oil occurred under reflux (30 min) using a potassium hydroxide solution in methanol. After proper treatment and pH adjustment, the methylation of free fatty acids was conducted with methanol under acidic catalysis, resulting in the formation of the corresponding methyl esters [17].

The analysis of the methyl esters was carried out using a gas chromatograph coupled to a mass spectrometer (GC‐MS) of the Shimadzu QP2010 series from Shimadzu Scientific Instruments Inc. (Columbia, MD, USA), employing a fused silica capillary column SH‐Rtx‐5 (30 m × 0.25 mm internal diameter; film thickness of 0.25 µm). The temperature program started at 80°C, increasing to 180°C at a rate of 4°C/min, followed by an increase to 246°C at a rate of 3.4°C/min, then reaching 280°C at 6.6°C/min, and finally at 10°C/min, totaling 30 min of analysis. The carrier gas flow rate was 1.5 mL/min, with injection in split mode (1:100) and an injection port temperature set to 220°C.

The operational parameters of the quadrupole mass spectrometer included an interface temperature of 280°C, an ion source temperature of 200°C, electron impact ionization at 70 eV, and a mass scan range of 40–350 m/z, with a sampling rate of 1.0 scan/s. The injection was performed with 1 µL of a 500‐ppm solution prepared in dichloromethane. The identification of constituents was conducted through a search in digital mass spectrum libraries (NIST 08) and comparison with reference spectra available in the literature [18].

### 2.4 In Silico Analysis

The investigation of the in silico properties of the major compounds (concentration above 30%) was conducted for palmitic acid (The Simplified Molecular Input Line Entry System (SMILES) is [CCCCCCCCCCCCCCCC(═O)O]) and elaidic acid (SMILES is [CCCCCCCC/C═C/CCCCCCCC(═O)O]) using the ADMETlab 2.0 platform. This tool is widely recognized for its advanced functionalities in analyzing parameters related to ADMET (absorption, distribution, metabolism, excretion, and toxicity), as well as physicochemical properties and medicinal chemistry aspects. The platform is based on more precise and efficient predictions, incorporating comprehensive analysis tools such as the toxicological interaction radar [19].

### 2.5 Statistical Analysis

The results of the experiments for minimum inhibitory concentration, antibiotic modifying were expressed as geometric mean ± standard error of the mean, evaluated statistically by analysis of variance (one‐way ANOVA) followed by Bonferroni post‐test using GraphPad Prism software. Differences were considered significant when *p* < 0.05.

## 3 Results

### 3.1 Phytochemical Profile

According to Araújo (2025), who used the same material as the present study, the fixed oil of *M. flexuosa* presented in its composition 18 fatty acids, most of which are saturated fatty acids, with a higher prevalence of elaidic (42.71%) and palmitic (37.07%) acids, and lower prevalence of acids such as stearic, linoleic, caprylic, valeric, suberic, azelaic, and pelargonic acids [20].

It is important to highlight that the derivatization method employed in this study (fatty acid methyl ester—FAME analysis) is specifically designed to identify fatty acids, which explains the predominance of these compounds in the chromatographic profile. Therefore, other classes of bioactive compounds, such as phenolic constituents and terpenoids, may not have been detected under the applied analytical conditions Table 1.

**TABLE 1:** Phytochemical composition of fixed oil of *Mauritia flexuosa* L.F. fruit.

Name	Time of retention (min)	%
Palmitic acid	15.632	37.07
Elaidic acid	17.952	42.71
Stearic acid	18.284	2.05
Linoleic acid	20.261	6.76
1,22‐Docosanediol	20.924	6.80
**Total**		**95.39**

### 3.2 Direct Antibacterial Activity

When analyzing the minimum inhibitory concentration of OFFMF against standard and multiresistant bacteria of *S. aureus* 10 and *P. aeruginosa* 24, MIC results ≥ 1024 µg/mL were obtained, not exhibiting inhibitory activity under these conditions. Nobre, 2018 [21] and Pereira, 2018 [22] obtained similar results when evaluating the antimicrobial activity of buriti fruit pulp oil against Gram‐positive and Gram‐negative bacteria.

### 3.3 Modifying Effect of Antibiotic Action

The modifying effect of OFFMF on the antibiotic activity was tested in combination with the antibiotics: erythromycin, gentamicin and norfloxacin with the aim of evaluating an interaction between them, in order to observe a possible potentiating effect against the multiresistant strains *S. aureus* 10 and *P. aeruginosa* 24.

**FIGURE 1:**
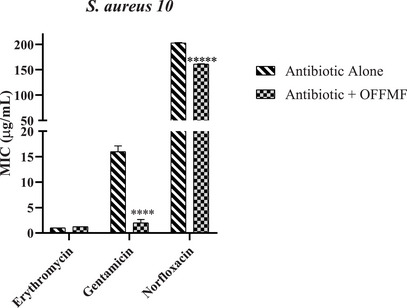
Association of OFFMF with the antibiotics erythromycin, gentamicin and norfloxacin against the *S. aureus* 10 strain. MIC values (µg/mL); OFFMF—Fixed oil from the fruit of *M. flexuosa*. Data expressed as mean ± SEM (n = 3). One‐way ANOVA followed by Bonferroni test. ****p < 0.0001 versus control.

When analyzing the results shown in Figure 1, it was found that OFFMF, when associated with erythromycin against the *S. aureus* 10 strain, presented nonsignificant results in relation to the control, that is, the oil did not present a potentiating effect for the tested strain. However, when associated with gentamicin, the compound presented potentiating the action of the antibiotic, reducing the MIC from 16 µg/mL to 2 µg/mL. The same effect was observed when the oil was associated with norfloxacin, with a reduction in the MIC from 203 µg/mL to 161.2 µg/mL.

**FIGURE 2:**
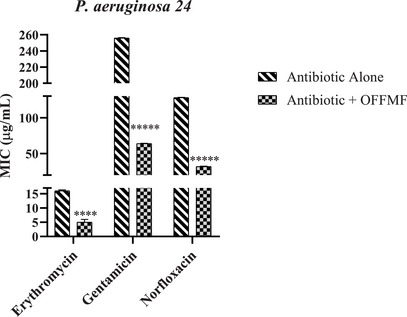
Association of OFFMF with the antibiotics erythromycin, gentamicin and norfloxacin against *P. aeruginosa 24* strain. MIC values (µg/mL); OFFMF—Fixed oil from the fruit of *M. flexuosa*. Data expressed as mean ± SEM (n=3). One‐way ANOVA followed by Bonferroni test. ****p < 0.0001 versus control.

Regarding the association of OFFMF with the antibiotics used against the *P. aeruginosa* strain, as demonstrated in Figure 2, it was possible to observe the reduction of the MIC, in association with erythromycin from 16 µg/mL to 5 µg/mL, gentamicin from 256 µg/mL to 64 µg/mL and norfloxacin from 128 µg/mL to 32 µg/mL.

### 3.4 Evaluation of Increased Bacterial Membrane Permeability

It was observed that OFFMF 200 µg/mL and 100 µg/mL increased the fluorescence intensity of SYTOX Green compared to the negative control, against S. aureus and E. coli strains. Against *S. aureus*, the oil increased fluorescence by 60% and 58%, respectively, at concentrations of 200 µg/mL and 100 µg/mL, compared to the negative control PBS.

**FIGURE 3:**
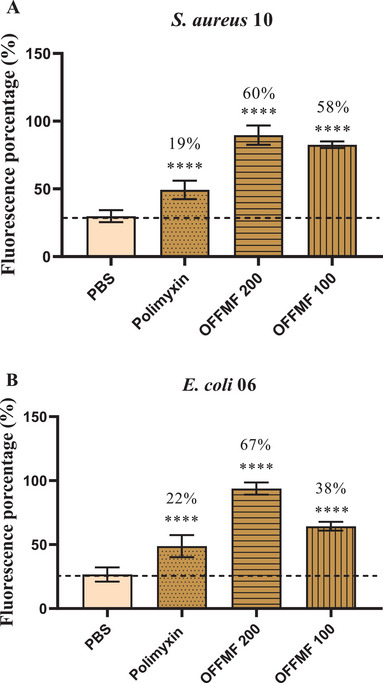
Evaluation of the action of Fixed Oil of *Mauritia flexuosa* (OFFMF) at 200 µg/mL and 100 µg/mL on the permeability of the bacterial membrane of *S. aureus* 10 (A) and *E. coli* 06 (B). ****p < 0.0001 versus PBS (negative control). Results are expressed as percentage of fluorescence intensity.

Against *E. coli*, the oil increased fluorescence by 67% and 38%, respectively, at concentrations of 200 µg/mL and 100 µg/mL. The results indicate that the fixed oil affects membrane permeability. The positive control polymyxin B at 200 µg/mL significantly increased the fluorescence intensity of SYTOX Green in both strains tested (Figure 3A,B).

### 3.5 Prediction of Elaidic and Palmitic Acid

The selection of the major compounds used for the in silico analyses was based on a previously published phytochemical characterization of OFFMF [20], in which a broader range of constituents was identified. In the present study, only the predominant compounds were considered in order to focus on the main contributors to the biological activity.

**FIGURE 4:**
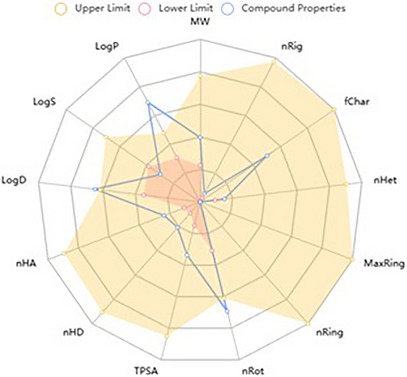
Radar of physicochemical characteristics of palmitic acid, comparison of the desirable minimum (lower) and maximum (upper) measurement.

**FIGURE 5:**
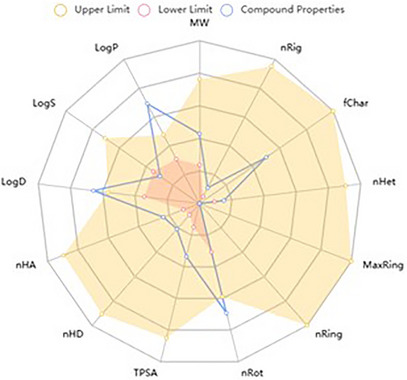
Radar of physicochemical characteristics of elaidic acid, comparison of the desirable minimum (lower), and maximum (upper) measurement. MW: molecular weight; nRig: Number of rigid bonds; fChar: Formal charge; nHet: Number of heteroatoms; MaxRing: Number of atoms in the biggest ring; nRing: Number of rings; nRot: Number of rotatable bonds; TPSA: Topological Polar Surface Area; nHD: number of hydrogen donos; nHA: number of hydrogen acceptors; logP: Log of the octanol/water partition coeficiente; logD: logP at physiological pH 7.4; LogS: Log of the aqueous solubility.

The in silico evaluation of the predominant compounds in the fixed oil of *M. flexuosa* indicates that palmitic and elaidic acids exhibit structural characteristics compatible with pharmaceuticals. As shown in the physicochemical property radar (Figures 4 and 5), these substances share similarities in terms of the number of hydrogen bond acceptors (nHA) and donors (nHD). However, some properties exceed the recommended limits, such as the aqueous partition coefficient (LogP > 3), distribution at physiological pH (LogD > 3), and the number of rotatable bonds (nRot = 0∼11), which may impact molecular flexibility.

Table 2 presents the similarity of the compounds to pharmaceuticals, showing that neither fully meets the criteria established by Pfizer's rules (logP > 3; Topological Polar Surface Area (TPSA) < 75) and GSK's rules (MW ≤ 400; logP ≤ 4). However, they remain compatible with Lipinski's and Golden's criteria. Considering these parameters is essential for identifying potential toxic compounds.

**TABLE 2:** Physicochemical properties and similarity with drugs, in silico analysis of elaidic and palmitic acid.

Category	Property	Fatty acid	
		Elaidic acid	Palmitic acid
Physicochemical Property	MF	C_18_H_34_O_2_	C_16_H_32_O_2_
	MW	282.260	256.240
	NHA	2	2
	NHD	1	1
	NRB	15	14
	Flexibility	7.500	14.000
	TPSA	37.300	37.300
	logS	−4.628	−5.223
Medicinal Chemistry	Lipinski Rule	Accepted	Accepted
	Pfizer Rule	Rejected	Rejected
	GSK Rule	Rejected	Rejected
	Golden Triangle	Accepted	Accepted

MF: molecular formula; MW: molecular weight; NHA: number of hydrogen acceptors; NHD: number of hydrogen donors; NRB: number of rotatable bonds; TPSA: Topological Polar Surface Area; LogS: Log of the aqueous solubility.

The properties related to absorption, distribution, excretion, metabolism, and toxicity (ADMET) of these fatty acids are detailed in Table 3. It is observed that human intestinal absorption (HIA) values are low for both compounds, and they do not act as substrates or inhibitors of permeability glycoprotein (Pgp), a protein involved in active cellular efflux. Another positive aspect observed is their low permeability across the blood‐brain barrier (BBB). The interaction with CYP complex isoenzymes is also noteworthy, as the inhibition of these enzymes can lead to drug interactions by influencing the metabolism of chemical compounds.

**TABLE 3:** ADME/Tox (absorption, distribution, metabolism, excretion, and toxicity) performance of elaidic and palmitic acid.

Category	Property	Fatty acid (Value)	
		Elaidic acid	Palmitic acid
Absorption	Caco‐2 Permeability	−5.096	−5.027
	MDCK Permeability	3e–05	2.5e–05
	Pgp‐inhibitor	**−**	**−**
	Pgp‐substrate	**−**	**−**
	HIA	**−**	**−**
Distribution	BBB Penetration	**−**	**−**
Metabolism	CYP1A2 inhibitor	−	−
	CYP2C19 inhibitor	−	−
	CYP2C9 inhibitor	−	−
	CYP2D6 inhibitor	**−**	**−**
	CYP3A4 inhibitor	**−**	**−**
Excretion	CL	2.414	2.377
	T_1/2_	0.768	0.610
Toxicity	Human Hepatotoxicity	**−**	**−**
	Drug Induced Liver Injury	**−**	**−**
	AMES Toxicity	**−**	**−**
	Rat Oral Acute Toxicity	**−**	**−**
	Skin Sensitization	**+++**	**++**
	Carcinogencity	**−**	**−**
	Eye Corrosion	**+++**	**+++**
	Eye Irritation	**+++**	**+++**
	Respiratory Toxicity	**++**	**++**

P‐gp: p glycoprotein; HIA: Human intestinal absorption; BBB: Blood–Brain Barrier; CYP: Cytochrome‐P; CL: Clearance; T_1/2_: Half‐life time. Symbols means 0–0.1 (−), 0.1–0.3 (–), 0.3–0.5 (‐),0.7–0.9 (++) e 0.9–1.0 (+++); (−) Nontoxic; (+++) high possibility of toxic activity.

The toxicological profile, summarized in Tables 3 and 4, indicates a low toxic potential for these compounds, except in dermatological, ophthalmological, and respiratory applications, where there is a possibility of adverse effects such as irritation and sensitization. Additionally, as described in Table 4, both compounds may interact with the peroxisome proliferator‐activated receptor gamma (PPAR‐γ). Elaidic acid, in particular, exhibits more pronounced toxicological characteristics, with the potential to induce toxicity by compromising mitochondrial membrane potential.

**TABLE 4:** Possible molecular targets of toxicity of elaidic and palmitic acids.

Property	Fatty acid (Value)	
	Elaidic acid	Palmitic acid
Androgen receptor	**−**	−
Androgen receptor ligand‐binding domain	**−**	**−**
Aryl hydrocarbon receptor	**−**	**−**
NR‐Aromatase	**−**	**−**
Estrogen receptor	−	−
Estrogen receptor ligand‐binding domain	**−**	**−**
Peroxisome proliferator‐activated receptor gamma	**+++**	**+++**
Antioxidant response element	−	−
ATPase family AAA domain‐containing protein 5	**−**	**−**
Heat shock factor response element	**+**	−
Mitochondrial membrane potential	**++**	−

(−) and (–): Nontoxic feature; (‐): Not very relevant value; (++) and (+++): high possibility of toxic activity.

It is important to emphasize that the ADMET predictions were obtained using in silico computational tools and should therefore be interpreted as theoretical estimations rather than experimental validation. Although these approaches are valuable for preliminary screening, further in vitro and in vivo studies are required to confirm the pharmacokinetic and toxicological profiles of the identified compounds.

## 4 Discussion

Nobre et al. and Silva et al. [21, 23] identified oleic and palmitic acids as the main constituents of the oil. Similarly to these studies, Pereira et al. [22] also found oleic acid (89.81%) and palmitic acid (10.19%) in larger quantities. A study carried out using the ethanolic extract of buriti leaves found the presence of phthalic and palmitic acids [24]. The difference in the geographical origin of collection, seasonality, and extraction method can explain the difference in compounds present in the oil [25].

The presence of fatty acids is common in fixed oils of the Arecaceae family, according to the study by [26], in the oils from the pulp and almond of *Syagrus oleraces, Syagrus romanzoffiana, and Acrocomia aculeata*, high concentrations of palmitic and oleic acid were found. Such compounds were also found in the oils of *Caryocar coriaceum* [27] and *Attalea speciosa* [28].

The study by Batista (2012), carried out using the agar diffusion method demonstrated antibacterial activity of buriti oil against strains of *S. aureus*, presenting an inhibition halo of 11.1 mm [11]. The study carried out by [29] with ethanolic and hexane extracts of buriti fruits showed a highly inhibitory effect of the extracts against *S. aureus* and *P. aeruginosa*.

The divergence between the results of this and other studies may be related to the method used to determine antibacterial activity, the phenotypic profile of the strains used, as well as the geographic region and environmental characteristics to which the plants were exposed, which could affect the biological activities of the plant [30].

When we observe the results of the antibiotic potentiation effect demonstrated in this study, we see that the data corroborate with [22] obtained a synergistic effect of buriti oil on *S. aureus* 10 with a reduction in the MIC of gentamicin and amikacin by 40.00% and 60.55%, respectively. The study by [31] using bioactive compounds from the buriti fruit extracted by ethanol and a supramolecular solvent system (SUPRAS), also demonstrated that the modulating activity of the supra bark extract and supra pulp extract of the buriti fruit, potentiated the action of the antibiotics norfloxacin and gentamicin against *S. aureus* 10, *P. aeruginosa* 24 and *E. coli* 06.

The study carried out by [32] also revealed modulating activity, using gentamicin combined with crude buriti oil and a nanoencapsulation of buriti oil (OPG) for *S. aureus*; the authors obtained an antagonism characterized by a 100% increase in MIC in both cases demonstrated. Still on the results of the study by [32] when evaluating norfloxacin alone, when compared with norfloxacin combined with crude buriti oil, the authors found that there was no modulating activity for *S. aureus* with a statistically significant difference (*p* > 0.05); however, the antibiotic activity combined with OPG decreased the MIC by 50%.

The study by [32] also showed a potentiating effect, the association between norfloxacin combined with buriti oil and an encapsulation of buriti oil (OPG) had greater inhibition for *P. aeruginosa* than norfloxacin alone, with a statistical difference between them (*p* < 0.05) and a 99% reduction in MIC with OPG and 97% with crude buriti oil, in addition, gentamicin showed a significant difference in the two combinations (with free oil and OPG), in which there was a 50% reduction in MIC.

In the study carried out by [31], an antagonistic effect was observed in the association of the bark extract of buriti fruits with gentamicin against *P. aeruginosa* 24. According to the author, one of the mechanisms that can explain this antagonistic effect is the chelation of the antibiotic by constituents of the extracts, thus inhibiting its action. When the same extracts were associated with norfloxacin, there was a potentiation of the antibiotic activity.

OFFMF showed better results for *S. aureus* when combined with gentamicin than when combined with erythromycin or norfloxacin. For *P. aeruginosa*, the compound showed very significant antibiotic modification values, showing a reduction in the MIC for all antibiotics tested. No data were found in the literature on the association of OFFMF and erythromycin. However, despite the nonsignificant data obtained with the association of OFFMF and erythromycin against *S. aureus*, the same compound potentiated the action of the antibiotic against *P. aeruginosa*, with a reduction in the MIC of 68.75%. These results demonstrate that the modulating effect of the oils can vary depending on the antibiotic and the bacterial strain tested [33].

The results obtained in this study can be attributed to the major constituents of buriti oil, mainly fatty acids. According to [34, 35], the presence of fatty acids in fixed oils helps their ability to act as antibacterials or antibiotic modifiers, since it has already been demonstrated that some fatty acids are capable of improving the antimicrobial effect.

The antibacterial activity of fatty acids may be related to their detergent property due to their amphipathic structure, thus allowing interaction with the lipid bilayer of the cell membrane, affecting metabolic processes essential for the acquisition of energy by the bacterial cell, such as the electron transport chain, oxidative phosphorylation and nutrient absorption. [7, 22, 35].

According to [11], the antibacterial effect of plant‐based products is more intense on Gram‐positive bacteria than on Gram‐negative bacteria, due to the peculiarities of the structural conformation of the cell wall of these bacteria. OFFMF did not show relevant intrinsic antibacterial activity; however, it demonstrated modulatory effects when combined with antibiotics in both Gram‐positive and Gram‐negative strains. Therefore, it is possible to conclude that even though no relevant results were found for the isolated use of OFFMF as an antibacterial agent, the compound, when associated with antibiotics, was able to reduce the MIC of the drugs tested, thus reducing the dose required to inhibit multiresistant microorganisms.

Several fatty acids exhibit antibacterial activity, acting through different mechanisms that justify their effects in bacterial control, primarily targeting the cell membrane. Among the identified mechanisms of action, the disruption of the electron transport chain, oxidative phosphorylation, and leakage of cellular metabolites stand out, interfering with essential processes for cellular protection and function [7]. This behavior is associated with the amphipathic properties of these molecules, which promote destabilization and compromise the integrity of the cell membrane [36].

Among the most relevant fatty acids with antibacterial activity, palmitic acid (hexadecanoic acid, C16:0) [36, 37] has been identified as a compound with pharmacological and biological potential, demonstrating activity against *E. coli* and *S. aureus* [37]. This activity can be explained by its molecular structure, which allows for good absorption in the body, enabling its use in oral formulations, as evidenced by HIA tests and Lipinski's Rule [38, 39, 40]. The intestinal absorption of this compound leads to a cascade effect, as it enters the bloodstream, is distributed to different tissues, and crosses cell membranes, allowing interaction with target receptors [41].

Regarding *Escherichia coli*, palmitic acid has been identified as a potential antibacterial agent due to its interaction with and inhibition of the enoyl‐acyl carrier protein reductase (FabI), a key target for antibacterial compounds [42]. Additionally, this compound can trigger different apoptotic mechanisms, including increased reactive oxygen species (ROS) production, mitochondrial dysfunction, endoplasmic reticulum stress, and autophagy induction [43, 44, 45, 46, 47, 48].

Another relevant fatty acid is the trans‐unsaturated elaidic acid (trans‐9‐octadecenoic acid), which has been studied for its molecular dynamics properties [49]. Evidence suggests that this compound significantly interacts with the IL‐1 receptor, the Nrf2 transcription factor, and peroxisome proliferator‐activated receptor α (PPAR‐α) proteins, indicating potential hepatoprotective and antioxidant effects [50]. Additionally, studies suggest a possible application of this fatty acid in colorectal cancer treatment due to its potential anticancer effects [51].

## 5 Conclusions

The present study demonstrated that the fixed oil from *Mauritia flexuosa* does not exhibit significant intrinsic antibacterial activity under the tested conditions; however, it showed a relevant modulatory effect when combined with conventional antibiotics. This effect may be associated with increased bacterial membrane permeability, as suggested by the SYTOX Green assay, indicating a possible mechanism involving disruption of membrane integrity. These findings reinforce the potential of natural products as adjuvants capable of enhancing antibiotic efficacy, especially against resistant bacterial strains. The chemical composition of the oil, predominantly consisting of fatty acids, may contribute to this biological activity, particularly due to their known interactions with lipid bilayers. The ADMET predictions suggested a generally favorable pharmacokinetic and toxicity profile, although these results should be interpreted cautiously, as they are based on computational models. Overall, this study contributes to the understanding of the modulatory effects of *M. flexuosa* oil and supports its potential application as an adjuvant in antimicrobial therapy.

## Author Contributions

**Conceptualization, Isaac Moura Araújo, Cleidiana Tomé de Oliveira, Luís Pereira‐de‐Morais**: methodology, **Andressa Silva Alencar, Maria Alícia Cavalcante Narciso, Anita Oliveira Brito Pereira Bezerra Martins, Sara Tavares de Sousa Machado, Sheila Alves Gonçalves, Daniel Sampaio Alves**: software, **Cicera Datiane de Morais Oliveira‐Tintino, José Thyálisson da Costa Silva**.: validation, **Isaac Moura Araújo**.: formal analysis, **Janaina Esmeraldo Rocha; investigation, Ana Carolina Ferreira Araújo**.: resources, **José Weverton Almeida‐Bezerra, Priscilla Ramos Freitas**.: writing−review and editing, **Ana Carolina Ferreira Araújo**.: supervision, **Henrique Douglas Melo Coutinho**.: funding acquisition, **Henrique Douglas Melo Coutinho**. All authors have read and agreed to the published version of the manuscript.

## Funding

This work received no external funding.

## Conflicts of Interest

The authors declare no conflicts of interest.

## Data Availability

The data that support the findings of this study are available on request from the corresponding author (IMA).
